# Prognostic nutritional index as a predictor of survival in resectable gastric cancer patients with normal preoperative serum carcinoembryonic antigen levels: a propensity score matching analysis

**DOI:** 10.1186/s12885-018-4201-4

**Published:** 2018-03-13

**Authors:** Noriyuki Hirahara, Yoshitsugu Tajima, Yusuke Fujii, Shunsuke Kaji, Tetsu Yamamoto, Ryoji Hyakudomi, Takahito Taniura, Yasunari Kawabata

**Affiliations:** Department of Digestive and General Surgery, Shimane University Faculty of Medicine, 89-1 Enya-cho, Izumo, Shimane 693-8501 Japan

**Keywords:** Gastric cancer, Prognostic nutritional index, Normal CEA, Prognostic factor

## Abstract

**Background:**

An ideal tumor marker should be capable of being detected at any stage of the disease. However, gastric cancer patients do not always have elevated serum carcinoembryonic antigen (CEA) levels, even in advanced cases. Recently, several studies have investigated the associations between preoperative PNI and postoperative long-term outcomes. In this study, we focused on the significance of the prognostic nutritional index (PNI) as a potential predictor of survival in resectable gastric cancer patients with normal preoperative serum CEA levels.

**Methods:**

We retrospectively conducted cohort study to evaluate the PNI as a predictor of survival in 368 resectable gastric cancer patients who underwent potentially curative gastrectomy at our institute between January 2010 and December 2016. We selected 218 patients by propensity score matching to reduce biases due to the different distributions of co-variables among the comparable groups.

**Results:**

In the multivariate analysis, pStage (hazard ratio [HR]: 14.003, 95% confidence interval [CI]: 5.033–44.487; *p* <  0.001), PNI (HR: 2.794, 95% CI: 1.352–6.039; *p* <  0.001) were identified as independent prognostic factors of CSS in 218 propensity matched gastric cancer patients. The Kaplan-Meier analysis demonstrated that low PNI patients had a significantly poorer cancer specific survival (CSS) than high PNI patients (*p* = 0.008).

Among 166 propensity matched gastric cancer patients with normal preoperative serum CEA levels, multivariate analysis demonstrated that pStage (HR: 7.803, 95% CI: 3.015–24.041; *p* <  0.001) and PNI (HR: 3.078, 95% CI: 1.232–8.707; *p* = 0.016) were identified as independent prognostic factors of CSS. And Kaplan-Meier analysis demonstrated that low PNI had a significantly poorer CSS than high PNI value (*p* = 0.011).

**Conclusions:**

This study demonstrates that a low preoperative PNI value is a potential independent risk factor for poorer CSS in patients with gastric cancer, even in those with normal serum CEA levels.

## Background

In recent years, there has been increasing concern regarding the association between the systemic inflammatory response and survival in patients with various types of cancer [1–3]. In addition, the systemic inflammatory response has attracted considerable attention as a unique prognostic factor independent of conventional tumor markers [4, 5]. Most tumor markers are produced by tumor cells. However, systemic inflammation involves biochemical reactions in response to cancer cell secreted inflammatory cytokines [6]. Cancer-related inflammatory changes induced by hypercytokinemia can be indirectly evaluated using multiple assessment tools (e.g., Glasgow prognostic scores, neutrophil-to-lymphocyte ratios, and lymphocyte-to-monocyte ratios) [7, 8]. Several studies have investigated the associations between preoperative nutritional status, cancer-related inflammation, and postoperative long-term outcomes. However, there is little data on the impact of long-term outcomes in patients undergoing curative laparoscopic gastrectomy for gastric cancer.

An ideal tumor marker should be capable of being detected at any stage of the disease. However, a diagnosis of cancer cannot be made on the basis of tumor markers alone, because the majority of tumor markers lack sufficient sensitivity and specificity. Moreover, in clinical practice, it is not unusual to examine the normal range of tumor markers, even in patients with advanced cancer. Therefore, the establishment of an independent and complementary prognostic indicator other than conventional tumor markers is of great clinical significance. The prognostic nutritional index (PNI) was originally reported as a nutritional assessment tool for predicting the risk of operative morbidity and mortality after gastrointestinal surgery. Only recently has it been identified as an indicator of cancer-related systemic inflammation [9].

In this study, we examine the utility of the PNI as a predictor of survival in the propensity score matched gastric cancer patients with normal preoperative serum carcinoembryonic antigen (CEA) levels.

## Methods

### Patients

We retrospectively reviewed a database of medical records from 368 consecutive patients who had undergone potentially curative gastrectomy with R0 resection for histologically confirmed gastric adenocarcinoma at our institute between January 2010 and December 2016. We performed propensity score matching using R version 3.1.3 software to reduce biases due to the different distributions of co-variables among the comparable groups; grouping variable was depth of tumor, lymph node metastasis, and pathological stage.

R0 resection was defined as a complete resection without any microscopic resection margin involvement. Laparoscopic or laparoscopy-assisted gastrectomy was performed in all patients. The extent of gastrectomy and lymph node dissection was in accordance with the Japanese Gastric Cancer Treatment Guidelines (Version 3) [10]. The patients’ clinical characteristics, laboratory data, treatment, and pathological data were obtained from medical records. Among the patients with gastric adenocarcinoma, 166 had normal preoperative serum CEA levels (< 5 ng/ml).

All participants provided informed written consent. This study’s retrospective design was approved by the Institutional Review Board Committee and was in accordance with the Helsinki Declaration.

### Preoperative nutritional parameters

All laboratory data used for calculating preoperative nutritional parameters were obtained within 1 week before surgery. The following items were selected as concise constitutional evaluation methods: body mass index (BMI) = body weight (kg)/height (m^2^) and the PNI = 10 × serum albumin (g/dl) + 0.005 × total lymphocyte count (/mm^3^) in peripheral blood [9].

**Fig. 1:**
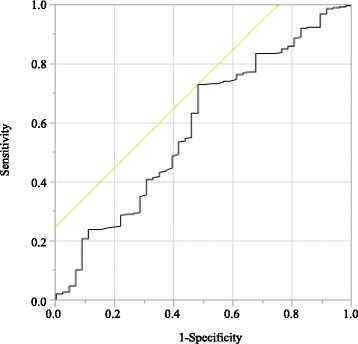
Receiver operating curves for post-operative survival were plotted to verify the optimum cut-off value of PNI for cancer-specific survival

A receiver operating characteristic curve of the preoperative PNI was generated for the multiple logistic regression analysis of cancer-specific survival (CSS). The area under the curve estimation method was used to assess the ability of the PNI to predict CSS. The optimal cutoff value of the PNI was set at 44.3, based on the 5-year postoperative CSS (Fig. 1) (sensitivity, 73.3%; specificity, 52.2%; and area under the receiver operating characteristic curve, 0.593). Patients were stratified into a high or low preoperative PNI group based on the cutoff value.

### Tumor staging

The pathological classification of the primary tumor, degree of lymph node involvement, and presence or absence of organ metastasis were determined according to the 7th edition of the American Joint Committee on Cancer TNM classification system [11].

### Statistical analyses

Quantitative variables were expressed as means ± standard deviation, while qualitative variables were expressed as numbers and percentages. Differences between the groups were evaluated using the Student’s *t*-test. Comparisons between non-normally distributed continuous variables among the three groups were performed using the Kruskal-Wallis test. Differences between the categorical variables were analyzed using the Chi-square test. CSS was calculated using the Kaplan-Meier method and compared by the log-rank test. CSS was defined as the time interval between the date of gastrectomy and the date of death from any cause, cancer-specific death, or withdrawal of consent.

Univariate analysis was performed to identify factors associated with CSS. Variables with a *p* <  0.05 in the univariate analysis were subjected to multivariate analysis using a Cox proportional hazards model to determine independent prognostic factors. Potential prognostic factors included age (< 70 vs. ≥ 70 years), sex (female vs. male), BMI (< 18.5 vs. ≥ 18.5), PNI (< 44.3 vs. ≥ 44.3), pathological Stage (pStage I/II vs. III), tumor size (< 5.0 vs. ≥ 5.0 cm), cancer cell differentiation (well- vs. moderately- and poorly-differentiated), preoperative serum CEA level (< 5.0 vs. ≥ 5.0 ng/ml), and postoperative adjuvant chemotherapy (“Yes” vs. “No”).

All statistical analyses were conducted using JMP software for Windows, version 11 (SAS Institute, Cary, NC, USA). All tests were two sided, and *p* <  0.05 was considered statistically significant.

## Results

### Associations between PNI values and clinicopathological characteristics in before and after propensity score matched patients

Associations between the PNI and clinicopathological characteristics of the entire cohort of 368 patients with gastric adenocarcinoma are summarized in Table 1. Based on a PNI cutoff of 44.3, 109 patients (29.6%) were included in the low PNI group and 259 patients (70.4%) were included in the high PNI group. PNI values were significantly associated with age (*p* <  0.001), BMI (*p* <  0.001), white blood cell counts (*p* = 0.004), red blood cell counts (*p* = 0.004), tumor size and depth (*p* <  0.001), lymph node metastasis (*p* = 0.010), pStage (*p* <  0.001), intraoperative blood loss (*p* = 0.009), serum albumin concentrations (*p* <  0.001), and C-reactive protein levels (*p* <  0.001).

**Table 1:** Relationships between PNI and clinicopathological features in overall gastric cancer patients before and after propensity score matching

		All patients				Propensity matched patients		
Characteristics	Total patients	PNI			Total patients	PNI		
		< 44.3	≥44.3			< 44.3	≥ 44.3	
		(*n* = 109)	(*n* = 259)	p value		(n = 109)	(*n* = 109)	*p* value
Age (years)		75.8 ± 9.2	68.5 ± 11.2	< 0.001		75.8 ± 9.2	68.3 ± 11.0	< 0.001
Sex				0.124				0.315
Male	254	69	185		145	69	76	
Female	114	40	74		73	40	33	
BMI		21.45 ± 3.42	22.85 ± 3.41	< 0.001		21.45 ± 3.42	22.61 ± 3.13	0.009
WBC (μl)		5416.8 ± 1464.3	5877.0 ± 1342.9	0.004		5416.8 ± 1464.3	6050.1 ± 1407.6	0.001
RBC (× 104 μl)		365.8 ± 55.9	465.1 ± 356.3	0.004		365.8 ± 55.9	470.4 ± 432.3	0.013
Location of tumor				0.187				0.333
EGJ	11	2	9		6	2	4	
U	70	23	47		41	23	18	
M	162	40	122		91	40	51	
L	125	44	81		80	44	36	
Tumor size (mm)		60.37 ± 33.40	41.89 ± 28.74	< 0.001		60.37 ± 33.40	48.06 ± 32.05	< 0.006
Procedure				0.090				0.954
LTG	82	31	51		60	31	29	
LPG	37	7	30		14	7	7	
L(A)DG	249	71	178		144	71	73	
Differentiation				0.123				0.919
Well	71	14	57		27	14	13	
Moderate	134	42	92		82	42	40	
Poor	163	53	110		109	53	56	
Depth of tumor				< 0.001				0.937
T1a-1b	190	40	150		80	40	40	
2	48	12	36		27	12	15	
3	54	23	31		45	23	22	
4a-4b	74	34	40		66	34	32	
Lymph node metastasis				0.010				0.813
N0	244	59	185		120	59	61	
N1	40	14	26		30	14	16	
N2	42	19	23		33	19	14	
N3	42	17	25		35	17	18	
Pathological stage				< 0.001				0.963
1a-1b	217	45	172		92	45	47	
2a-2b	65	26	39		51	26	25	
3a-3c	86	38	48		75	38	37	
Operation time (min)		416.1 ± 126.0	419.5 ± 116.3	0.804		416.1 ± 126.0	430.5 ± 121.2	0.391
Intraoperative								
blood loss (ml)		325.3 ± 699.0	180.8 ± 359.9	0.009		325.3 ± 699.0	257.0 ± 485.4	0.404
Albumin (g/dl)		3.26 ± 0.49	4.21 ± 0.34	< 0.001		3.26 ± 0.49	4.20 ± 0.35	< 0.001
CRP (mg/l)		0.705 ± 1.22	0.190 ± 0.540	< 0.001		0.705 ± 1.22	0.205 ± 0.478	< 0.001
CEA (ng/ml)				0.117				0.204
< 5	286	79	207		166	79	87	
> 5	82	30	52		52	30	22	
Adjuvant chemotherapy				0.541				0.035
Yes	100	32	68		79	32	47	
No	268	77	191		139	77	62	

Among high PNI patients 78.2% (68/87) with stage II and stage III received adjuvant chemotherapy, whereas low PNI patients only 50.0% (32/64) with stage II and stage III received adjuvant chemotherapy.

After propensity score matching, depth of tumor, lymph node metastasis, and pathological stage did not differ significantly between the low and high PNI groups. Finally, 218 patients were selected for analysis.

### PNI values and serum CEA levels in the propensity score matched patients

**Fig. 2:**
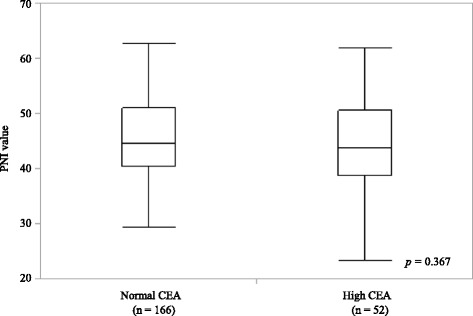
PNI values in propensity score matched 218 gastric cancer patients according to the serum level of carcinoembryonic antigen. Kruskal-Wallis test: *p* = 0.367. In each box plot, the lower and upper ends of the box represent the 25th and 75th percentiles, respectively. Capped bars indicate the minimum and maximum values, respectively, and the line inside the box represents the median PNI value

No association between the PNI values and serum CEA levels were detected using a one-way Kruskal-Wallis analysis (*p* = 0.367). The mean PNI values for patients with normal (*n* = 166) and elevated serum CEA levels (*n* = 52) were 45.34 ± 7.63 and 44.22 ± 8.11, respectively (Fig. 2).

### Cox regression analysis of CSS in the propensity score matched patients

In the univariate analysis, pStage (*p* <  0.001), tumor size (*p* = 0.025), PNI (*p* = 0.049), CEA level (*p* = 0.040), and adjuvant chemotherapy (*p* = 0.008) were significantly associated with CSS. In the multivariate analysis, pStage (hazard ratio [HR]: 14.003, 95% confidence interval [CI]: 5.033–44.487; *p* <  0.001), and PNI (HR: 2.794, 95% CI: 1.352–6.039; *p* <  0.001) were identified as independent prognostic factors of CSS in 218 propensity score matched patients (Table 2).

**Table 2:** Univariate and multivariate analyses to assess the prognostic factors in propensity score matched 218 gastric cancer patients

Variables		Univariate analysis			Multivariate analysis		
		HR	95% CI	*p* value	HR	95% CI	*p* value
Gender	female / male	1.414	0.690–3.116	0.353			
Age	< 70 / ≥ 70	0.709	0.355–1.410	0.325			
pStage	I, II / III	10.130	4.483–27.161	< 0.001	14.003	5.033–44.487	< 0.001
Tumor size	< 5 / ≥ 5	2.263	1.107–4.977	0.025	1.735	0.718–3.910	0.212
PNI	≥ 44.3 / < 44.3	2.000	0.999–4.197	0.049	2.794	1.352–6.039	< 0.001
CEA	< 5.0 / ≥ 5.0	2.136	1.036–4.254	0.040	1.821	0.878–3.649	0.105
Diff.	well & mod / poor	1.533	0.770–3.161	0.226			
BMI	≥ 18.5 / < 18.5	1.052	0.414–3.548	0.924			
Adjuvant	No / Yes	2.528	1.268–5.218	0.008	1.052	0.485–2.364	0.899

*HR* Hazard ratio, *CI* Confidence interval, *PNI* Prognostic nutritional index, *CEA* Carcinoembryonic antigen, *pStage* Pathological Stage, *Diff* Differentiation, *BMI* Body mass index, Adjuvant adjuvant chemotherapy

### Survival analysis stratified by the PNI in the propensity score matched patients

**Fig. 3:**
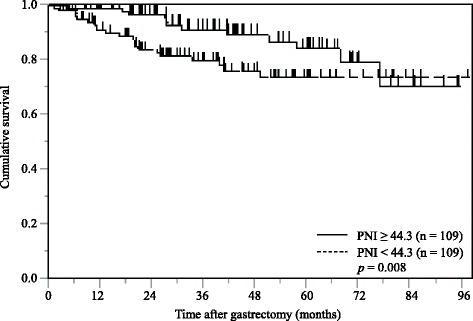
Kaplan-Meier curves of postoperative cancer-specific survival based on PNI in propensity score matched 218 gastric cancer patients

The Kaplan-Meier analysis and log-rank test demonstrated that patients with a low PNI value had a significantly poorer CSS than those with a high PNI value (*p* = 0.008; Fig. 3).

### Survival analysis stratified by the CEA in the propensity score matched patients

**Fig. 4:**
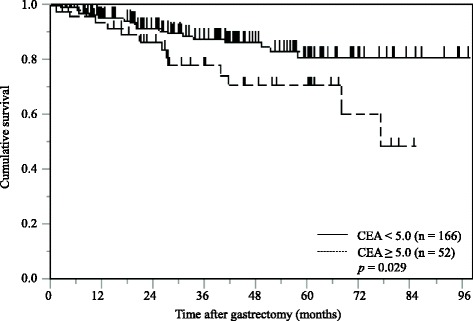
Kaplan-Meier curves of postoperative cancer-specific survival based on serum CEA levels in propensity score matched 218 gastric cancer patients

The Kaplan-Meier analysis and log-rank test demonstrated that propensity score matched 52 gastric cancer patients with a high serum CEA levels had a significantly poorer CSS than 166 patients with a low CEA levels (*p* = 0.029; Fig. 4).

### Associations between PNI values and clinicopathological characteristics in the propensity score matched patients with normal preoperative serum CEA levels

Associations between the PNI and clinicopathological characteristics of the 286 patients with normal preoperative serum CEA levels are summarized in Table 3. Based on a PNI cutoff of 44.3, 79 patients (27.6%) were included in the low PNI group and 207 patients (72.4%) were included in the high PNI group. PNI values were significantly associated with age (*p* <  0.001), BMI (*p* = 0.003), white blood cell counts (*p* = 0.009), red blood cell counts (*p* = 0.021), tumor size (*p* <  0.001), tumor depth (*p* = 0.001), lymph node metastasis (*p* = 0.032), pStage (*p* = 0.001), serum albumin concentrations (*p* < 0.001), and C-reactive protein levels (*p* < 0.001).

**Table 3:** Relationships between PNI and clinicopathological features in gastric cancer patients with normal serum CEA levels before and after propensity score matching

		All patients				Propensity matched patients		
Characteristics	Total patients	PNI			Total patients	PNI		
		< 44.3	≥44.3			< 44.3	≥ 44.3	
		(*n* = 79)	(*n* = 207)	p value		(n = 79)	(*n* = 87)	*p* value
Age (years)		75.2 ± 9.5	68.1 ± 11.5	< 0.0001		75.2 ± 9.5	68.2 ± 11.5	< 0.001
Sex				0.2912				0.347
Male	191	49	142		109	49	60	
Female	95	30	65		57	30	27	
BMI		21.45 ± 3.41	22.80 ± 3.39	0.0029		21.45 ± 3.41	22.54 ± 3.21	0.036
WBC (μl)		5367.0 ± 1482.3	5838.3 ± 1297.8	0.0088		5367.0 ± 1482.3	5916.4 ± 1259.0	0.011
RBC (× 104 μl)		368.4 ± 50.2	472.6 ± 397.5	0.021		368.4 ± 50.2	479.2 ± 483.3	0.044
Location of tumor				0.5422				0.634
EGJ	9	2	7		5	2	3	
U	48	12	36		26	12	14	
M	132	33	99		76	33	43	
L	97	32	65		59	32	27	
Tumor size (mm)		59.86 ± 31.41	41.06 ± 29.34	< 0.0001		59.86 ± 31.41	47.39 ± 32.81	0.014
Procedure				0.2763				0.872
LTG	59	19	40		43	19	24	
LPG	26	4	22		8	4	4	
L(A)DG	201	56	145		115	56	59	
Differentiation				0.2569				0.812
Well	53	10	43		20	10	10	
Moderate	106	33	73		66	33	33	
Poor	127	36	91		80	36	44	
Depth of tumor				0.0009				0.713
T1a-1b	154	31	123		64	31	33	
2	37	8	29		22	8	14	
3	41	16	25		33	16	17	
4a-4b	52	24	28		47	24	23	
Lymph node metastasis				0.0318				0.682
N0	199	46	153		98	46	52	
N1	31	9	22		23	9	14	
N2	30	12	18		24	12	12	
N3	26	12	14		21	12	9	
Pathological stage				0.001				0.884
1a-1b	178	36	142		76	36	40	
2a-2b	48	17	31		38	17	21	
3a-3c	60	26	34		52	26	26	
Operation time (min)		400.8 ± 116.0	415.6 ± 118.5	0.3418		400.8 ± 116.0	425.9 ± 119.4	0.172
Intraoperative								
blood loss (ml)		233.3 ± 388.3	163.0 ± 350.1	0.1421		233.3 ± 388.3	240.6 ± 486.0	0.915
Albumin (g/dl)		3.28 ± 0.48	4.21 ± 0.34	< 0.0001		3.28 ± 0.48	4.21 ± 0.36	< 0.001
CRP (mg/l)		0.682 ± 1.234	0.188 ± 0.555	< 0.0001		0.682 ± 1.234	0.191 ± 0.481	< 0.001
Adjuvant chemotherapy				0.4396				0.099
Yes	74	23	51		59	23	36	
No	212	56	156		107	56	51	

After propensity score matching, depth of tumor, lymph node metastasis, and pathological stage did not differ significantly between the low and high PNI groups. Finally, 79 low PNI patients and 87 high PNI patients were selected for analysis.

### Cox regression analysis of CSS in the propensity score matched patients with normal preoperative serum CEA levels

In the univariate analysis, pStage (*p* < 0.001), and PNI (*p* = 0.030), were significantly associated with CSS. In the multivariate analysis, pStage (HR: 7.803, 95% CI: 3.015–24.041; *p* < 0.001) and PNI (HR: 3.078, 95% CI: 1.232–8.707; *p* = 0.016) were identified as independent prognostic factors of CSS (Table 4).

**Table 4:** Univariate and multivariate analyses to assess the prognostic factors in propensity score matched 166 gastric cancer patients with normal serum CEA levels

Variables		Univariate analysis			Multivariate analysis		
		HR	95% CI	*p* value	HR	95% CI	*p* value
Gender	female / male	0.652	0.230–1.631	0.370			
pStage	I, II / III	7.303	2.827–22.468	< 0.001	7.803	3.015–24.041	< 0.001
Tumor size	< 5 / ≥ 5	1.735	0.710–4.618	0.230			
PNI	≥ 44.3 / < 44.3	2.742	1.100–7.745	0.030	3.078	1.232–8.707	0.016
Diff.	well & mod / poor	1.588	0.657–4.054	0.306			
BMI	≥ 18.5 / < 18.5	1.050	0.245–3.125	0.939			
Adjuvant	No / Yes	2.419	1.000–6.179	0.050			

*HR* Hazard ratio, *CI* Confidence interval, *PNI* Prognostic nutritional index, *CEA* Carcinoembryonic antigen, *pStage* Pathological Stage, *Diff* Differentiation, *BMI* Body mass index, Adjuvant adjuvant chemotherapy

### Survival analysis stratified by the PNI in the propensity score matched patients with normal preoperative serum CEA levels

**Fig. 5:**
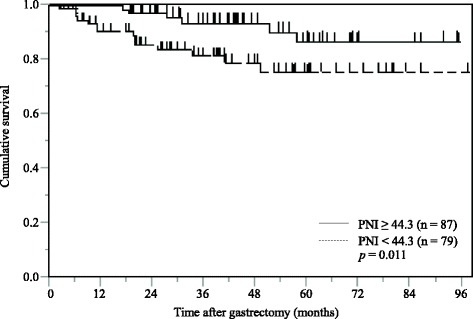
Kaplan-Meier curves of postoperative cancer-specific survival based on PNI in propensity score matched 166 gastric cancer patients with normal serum CEA levels

The Kaplan-Meier analysis and log-rank test demonstrated that patients with a low PNI value had a significantly poorer CSS than those with a high PNI value (*p* = 0.011; Fig. 5).

## Discussion

Diagnostic imaging modalities and several tumor markers, including preoperative ou. However, gastric cancer patients do not always have elevated serum CEA levels, even in advanced cases [12]. Moreover, serum CEA levels are more commonly used for postoperative surveillance. However, it cannot be expected to serve as a surrogate marker for cancer recurrence when the primary tumor exhibits normal serum CEA levels [13]. Therefore, in this study, we focused on the significance of the PNI as a potential predictor of survival in gastric cancer patients with normal preoperative serum CEA levels.

The PNI was initially developed to predict perioperative complications, such as anastomotic leakage, delayed tissue repair, and the length of postoperative hospital stay [9]. Recently, however, accumulating evidence suggests that the preoperative PNI could be a favorable prognostic factor and a more reliable assessment tool for the physiological status of cancer patients [14–16]. Albumin is a widely used nutritional parameter, and produced by hepatocytes and is regulated by pro-inflammatory cytokines, including inteleukin-1 (IL-1), IL-6, and tumor necrosis factor-α (TNF-α) that adversely affect catabolic metabolism. These proinflammatory cytokines are produced by the tumor itself or the host and play crucial roles in carcinogenesis, cancer progression, and neoangiogenesis [17, 18]. Similarly, lymphocytes are a fundamental component of the cytotoxic immune response that suppresses tumor cell proliferation and invasion via cytokine-mediated cytotoxicity [19, 20]. Hence, the PNI may represent a comprehensive indicator of the long-term prognosis of cancer patients.

First, we analyzed the associations between PNI values and the clinicopathological characteristics of 368 patients who underwent curative laparoscopic-assisted gastrectomy for Stage IA–IIIC gastric cancer. We demonstrated that a low preoperative PNI value was associated with age, BMI, white and red blood cell counts, a large tumor size, deep invasion, lymph node metastasis, an advanced pStage, lower albumin concentrations, and higher C-reactive protein levels, but not preoperative serum CEA levels. Our findings support the hypothesis that a low PNI value is indicative of chronic inflammation and malnutrition in patients with more aggressive or advanced cancers. In light of the significance of preoperative PNI values on survival, Kaplan-Meier analysis of the 368 patients in the entire cohort demonstrated that a low PNI value was associated with a significantly poorer CSS. In the multivariate analysis, a low PNI value was also confirmed to be a significant independent predictor of poor CSS. However, the precise mechanism underlying the association between PNI values and CSS has not been fully elucidated. On the other hand, several previous studies have reported that cancer patients experiencing postoperative complications generally have a poorer prognosis [21–23]. Because patients with a low preoperative PNI value are at a high risk of postoperative complications, the preoperative PNI value may affect both postoperative short- and long-term outcomes.

CEA is a glycoprotein attached to the surface of enterocytes, with a weight of 200 kDa and a role in programmed cell death and cell adhesion [24]. Although CEA is one of the most widely and frequently used tumor markers, especially in gastrointestinal cancer, its exact function in cancer screening, diagnosis, treatment decision-making, and postoperative surveillance remains poorly understood. Therefore, we evaluated the utility of the PNI as a predictor of survival in gastric cancer patients with normal preoperative serum CEA levels. We revealed that a low preoperative PNI value was a comprehensive indicator of cancer-related inflammation and a poor nutritional status in patients with normal serum CEA levels. Furthermore, the multivariate analysis demonstrated that a low PNI value was independently associated with a poor prognosis. These findings reflect the widely accepted hypothesis that the long-term outcome of cancer patients is not determined by tumor characteristics alone, but is also associated with cancer-related inflammation and malnutrition. In addition, several studies have reported that feasibility of adjuvant chemotherapy was defined by perioperative nutritional condition. Similarly, our result showed the ratio of feasibility of adjuvant chemotherapy is low in malnourished patients. Therefore, early pre- and post-operative nutritional support, through enteral feeds, early oral intake or intravenous feeding, has become an increasingly standard element of enhanced recovery care pathways following gastrectomy [25].

The significance of the PNI in cancer patients has not been uniformly confirmed, because the optimal cutoff point for the PNI in predicting postoperative survival remains controversial [26–28]. Thus, one of the aims of our study was to elucidate the optimal cutoff point of the preoperative PNI for predicting CSS in patients with gastric cancer. Based on receiver operating characteristic curve analysis of the 368 patients who had undergone curative gastrectomy, we determined an optimal cutoff value for the PNI of 44.3. This was remarkably close to the standard value of 45, reported by Onodera et al. [9], at which gastrointestinal anastomosis could be performed safely.

Our study has several limitations that need to be acknowledged. These include its uncontrolled and retrospective nature, single institutional design, relatively small sample size, and short follow-up period. Moreover, we excluded patients who had undergone neoadjuvant chemotherapy. Additionally, we focused on the preoperative PNI, but failed to evaluate dynamic changes in PNI values during the clinical course of the disease. Finally, the biological mechanisms associated with systemic inflammation and prognosis have yet to be elucidated. Therefore, further large-scale prospective studies are needed to determine the molecular mechanisms linking a low PNI value with a poorer prognosis in patients with gastric cancer.

Despite these limitations, we showed that a low preoperative PNI value is a potential independent risk factor for a poor prognosis in patients with gastric cancer, even in those with normal serum CEA levels. These results may be useful when considering the clinical decision-making process in gastric cancer patients with a low PNI.

## Conclusions

In this study, we confirmed that the PNI was associated with the CSS of gastric cancer patients after curative gastrectomy. It is particularly noteworthy that a low preoperative PNI value is a potential independent risk factor for poorer CSS in patients with gastric cancer, even in those with normal serum CEA levels. The PNI is convenient, cost effective and readily available, it could act as a marker of survival in gastric cancer. We offer evidence to show that an accessible parameter like PNI can help clinicians detect signs of recurrence very early and effectively customize treatment regimens.

## Abbreviations

Adjuvant: Adjuvant chemotherapy
BMI: Body mass index
CEA: Carcinoembryonic antigen
CI: Confidence interval
CRP: C-reactive protein
CSS: Cancer-specific survival
Diff: Differentiation
EGJ: Esophagogastric junction
HR: Hazard ratio
L: Lower
L(A)DG: Laparoscopic (assisted) distal gastrectomy
LPG: Laparoscopic proximal gastrectomy
LTG: Laparoscopic total gastrectomy
M: Middle
PNI: Prognostic nutritional index
pStage: Pathological Stage
RBC: Red blood cell
U: Upper
WBC: White blood cell
